# Isothermal microcalorimetry accurately detects bacteria, tumorous microtissues, and parasitic worms in a label-free well-plate assay

**DOI:** 10.1002/biot.201400494

**Published:** 2015-02-18

**Authors:** Olivier Braissant, Jennifer Keiser, Isabel Meister, Alexander Bachmann, Dieter Wirz, Beat Göpfert, Gernot Bonkat, Ingemar Wadsö

**Affiliations:** 1 Center for Biomechanics and Biocalorimetry, c/o Biozentrum-PharmazentrumBasel, Switzerland; 2 Department of Urology, University Hospital of BaselBasel, Switzerland; 3 Swiss Tropical and Public Health InstituteBasel, Switzerland; 4 Orthomerian, Basel, Switzerland; 5 Physical Chemistry, Chemical Center, Lund UniversityLund, Sweden

**Keywords:** Analytical biotechnology, Cell biology, Diseases, Isothermal microcalorimetry, Metabolic flux analysis

## Abstract

Isothermal microcalorimetry is a label-free assay that allows monitoring of enzymatic and metabolic activities. The technique has strengths, but most instruments have a low throughput, which has limited their use for bioassays. Here, an isothermal microcalorimeter, equipped with a vessel holder similar to a 48-well plate, was used. The increased throughput of this microcalorimeter makes it valuable for biomedical and pharmaceutical applications. Our results show that the sensitivity of the instrument allows the detection of 3 × 10^4^ bacteria per vial. Growth of *P. mirabilis* in Luria Broth medium was detected between 2 and 9 h with decreasing inoculum. The culture released 2.1J with a maximum thermal power of 76 μW. The growth rate calculated using calorimetric and spectrophotometric data were 0.60 and 0.57 h^–1^, respectively. Additional insight on protease activities of *P. mirabilis* matching the last peak in heat production could be gathered as well. Growth of tumor microtissues releasing a maximum thermal power of 2.1 μW was also monitored and corresponds to a diameter increase of the microtissues from ca. 100 to 428 μm. This opens new research avenues in cancer research, diagnostics, and development of new antitumor drugs. For parasitic worms, the technique allows assessment of parasite survival using motor and metabolic activities even with a single worm.

## 1 Introduction

All living organisms and many related biological processes produce heat. Calorimetric techniques can therefore, in principle, be used to perform many bioassays [1]. For example, calorimetry has recently been used for detection of infection [2–4], drug susceptibility testing [5, 6], screening for new drugs [7–10], food microbiology [11–14], material testing [15], and parasitology [16–18]. When such measurements are performed, it is the heat production rate (i.e., the thermal power) that is measured. This kinetic and thermodynamic property is related to the metabolic activity of the observed biological system. However, the relationship between the sum of metabolic processes and the obtained thermodynamic information is complex and the latter is often ignored. As a result, isothermal microcalorimeters are often used primarily as a “bio-activity” monitor.

As only heat production rate needs to be measured, calorimetric assays are label free and may be performed under all kinds of conditions with samples that have very different physical properties. For example, using calorimetry allows real-time monitoring of metabolic activity of cells in suspensions, solids, gels, or attached to a surface under oxic or anoxic conditions [1, 19, 20]. This makes isothermal microcalorimetry well suited for studying complex biological structures such as biofilms, tumor microtissues, or parasites hiding inside their host cells. For microtissues and some parasites such as parasitic worms, there are very few assays available [18, 21]. In addition, early calorimetric studies on biopsies and tumors were hindered by the lack of throughput of the instruments available in 1970s [22–24]. The same was true for studies on protozoans used as models for parasites or eukaryotic cells [25, 26]. Finally, the computing power at this time was not sufficient to analyze the power–time curves generated by worms using mathematical tools such as wavelets for example [17]. Thus, microscopy remained the gold standard for many parasitology and animal cell studies. However, microscopy is labor intensive and expensive for laboratories. Microcalorimetry has been shown to allow accurate measurement of worms metabolic and motor activity at very low workload [16–18] offering the potential to increase efficiency and decrease laboratory costs in drug screening for example.

Many types of calorimeters can be used for performing measurement of living systems, however isothermal microcalorimeters are now the most commonly used. The term isothermal microcalorimeter refers to calorimeters designed for measurements in the range of microwatt, or lower, under essentially isothermal conditions [27]. Most isothermal microcalorimeters in use are of the heat conduction type. In such instruments, the heat released in the reaction vessel is conducted through a thermopile plate before it is taken up by a surrounding heat sink. The sample volume ranges from 1 to 25 mL and time constants are usually in the order of a few min. The rate of heat flow through the thermopile is proportional to the thermopile potential. As a result, if the rate of the process is rather constant the potential will be proportional to the thermal power (the rate of heat production) that is released in the reaction vessel. If the change in reaction rate is fast compared to the time constant of the instrument, a more complex relationship must be used in order to compensate for the thermal inertia of the calorimeter [28]. The sensitivity of modern microcalorimeters is higher than for many conventional methods (spectrophotometry or enzymatic assays for example). They can detect the metabolic heat production of 10^4^–10^5^ bacteria, 10^3^–10^4^ protozoan, or 10^2^–10^3^ hepatocytes [25, 29, 30]. Multi-channel versions of such instruments allows the simultaneous measurements of many samples [1], thereby increasing the throughput, and makes the technique interesting as a bio-activity monitor for many biomedical and pharmaceutical applications. However, most commercial instruments available today still have low throughput (i.e., 1–12 channels). As an alternative, chip calorimeters [31–34] used in combination with flow-through systems can allow a much higher throughput than conventional isothermal microcalorimeters. Such chip calorimeters have recently been used as bio-activity monitors in exploratory studies [35, 36]. However, their small volume (10 nL to 100 μL) and their lower sensitivity compared to conventional instruments, limit their use with dilute cell suspensions and heterogeneous materials like tissue preparations and small animals.

In this context, recently developed well-plate isothermal microcalorimeters are of particular interest for biologists since well-plates are a classical format. In addition to taking advantage of the sensitivity and label-free nature of this technique, well-plate calorimeters offer a higher throughput than many conventional instruments [31, 33, 34], although at the cost of a slightly lower reaction volume. Still such low volume is sufficient not only for studies with microbes, but also for engineered microtissues, biopsies, or parasitic worms. Here, we investigate the performances of a prototype isothermal microcalorimeter with a well-plate format sample holder for use in bacteriology, oncology, and parasitology.

## 2 Materials and methods

### 2.1 Isothermal microcalorimeter

**Figure 1:**
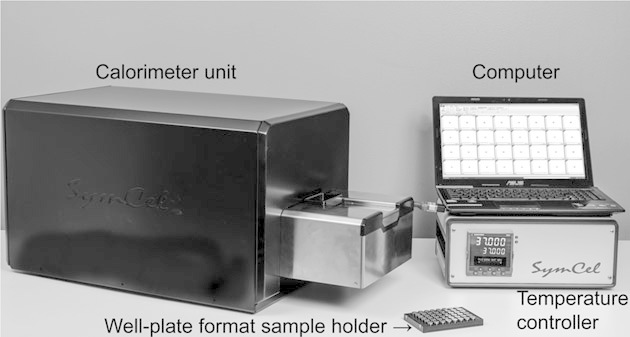
Picture of the instrument used for the study. The instrument is composed of a calorimeter unit maintained at constant temperature, a PID temperature controller, a computer to record the data and a well-plate format sample holder.

For all experiments we used a pre-production model of a 48-channel isothermal microcalorimeter (Symcel Sverige AB, Kista, Sweden – Fig. 1). In this instrument, 48 peltier elements are in thermal contact with an aluminum block that serves as heat sink. The construction is suspended in the cavity of a cuboid shaped block that is thermostated at the chosen experimental temperature. This assembly forming the calorimeter unit is positioned in a steel Dewar vessel, which is mounted in a horizontal position. A third aluminum block is positioned between the calorimeter and the lid closing the Dewar vessel. This block is also thermostated at the experimental temperature and serves as a pre-thermostat for the samples during their introduction into their measurement positions. The samples are contained in screw cap vials made from stainless steel or titanium that are closed by lids fitted with O-ring seals. Volume and inner diameter of the vials are 0.6 mL and 8 mm, respectively. The vials are positioned in a plastic well-plate format sample holder. The well-plate format sample holder is brought to the measurement position through a narrow horizontal channel passing through the thermostated aluminum blocks and the heat sink. A 3-step procedure is recommended by the manufacturer for thermal equilibration. In this procedure, the well-plate format sample holder stays between 10 and 15 min on the first two aluminum blocks. Then, it is pushed in the measurement position where it stays about 30 min before proper thermal equilibration is achieved. Usually 1 h was necessary before thermal equilibrium was achieved and measurements could be recorded.

Up to 47 samples can be measured simultaneously – one position in the plate is charged with an inert sample, which is used as a reference. For optimal performance at low power values the instrument can be operated with multiple separate reference vessels. In our measurements, 32 channels were used for samples as 16 were used as references (i.e., one reference for two neighboring samples). The thermostat was set at 37 °C for all experiments. At this temperature, the instrument has a short-term noise below 50 nW for most channels and a baseline drift below 100 nW per 24 h (manufacturer data for an empty instrument – note that adding vials usually increase the stability). However, we noticed that rapid changes in room temperature did affect the baseline stability. The microcalorimeter data were sampled at a frequency of 1 data point every 25 s and were stored as an ASCII file that could be edited in commonly used spreadsheet software.

### 2.2 Growth of *Proteus mirabilis (Deutsche Sammlung von Mikroorganismen und Zellkulturen, DSMZ 4479)*

The absorbance of an overnight culture was measured and the culture was further diluted to obtain an initial concentration of 10^8^ colony forming units (cfu). To determine the sensitivity of the instrument, serial dilutions were performed in order to achieve cell densities ranging from 10^6^ to 10^1^ cfu/mL. 300 μL of each dilution was placed in stainless steel microcalorimetric vials. The same procedure was repeated with medium containing 4 mg/L of chloramphenicol to avoid cell divisions during the thermal equilibration period of 1 h. We chose a sub-inhibitory concentration of chloramphenicol that inhibits growth by perturbing the synthesis of new proteins without affecting activities of those previously made and thus metabolic heat production.

To investigate the relationship between the thermal power curve and conventional parameter to estimate growth, serial dilutions were performed in order to achieve cultures with a cell density ranging from 10^6^ to 10^0^ cfu/mL. 300 μL of each dilution was placed in stainless steel microcalorimetric vials. The vials were then sealed and inserted in the well-plate microcalorimeter according to the manufacturer instructions. A parallel set of vials was prepared and placed in an incubator at 37 °C. Using this set of samples we validated the bacterial measurements by monitoring the OD using a Uvikon 860 spectrophotometer (Kontron, Eching, Germany). To further identify the processes happening during the stationary growth phase protease activity was quantified using the azocasein assay. Briefly, 150 μL of cell culture from a calorimetric vial were centrifuged and 100 μL of supernatants were incubated with 400 μL of 1% azocasein for 30 min at 37 °C. The reaction was stopped by adding 600 μL of 10% trichloro acetic acid further incubation on ice for 30 min. The samples were then centrifuged at 14 000 rpm for 5 min and 800 μL of the resulting supernatants were added to a polystyrene spectrophotometer cuvette containing 200 μL of 1.8 N sodium hydroxide. Absorbances at 420 nm of the samples were measured using a Uvikon 860 spectrophotometer (Kontron, Eching Germany). The amount of azo dye release by protease was calculated using the Beer–Lambert law assuming a molar absorption coefficient of 82 600 L/mol/cm.

### 2.3 Growth of tumor microtissues

Tumor microtissues were obtained from InSphero (Schlieren, Switzerland). In this study we used hepatocarcinoma microtissues based on the HepG2 cell line (American type culture collection, ATCC® HB-8065). Upon receipt, different numbers of microtissues were immediately transferred into titanium microcalorimetric vials by pipetting (according to the handling instructions of the provider). After transfer, the original medium was replaced with 300 μL of fresh 3D culture medium (InSphero 3D InSight™ Cell-Culture Media). The vials were then sealed and inserted in the well-plate microcalorimeter according to manufacturer instructions. The metabolic heat signal was recorded until it returned to baseline (i.e., until metabolic activity was no longer detectable). At the end of the culture, vials were reopened and the size of the microtissues was measured microscopically. Finally 10 μL of the culture medium were streaked on a brain heart infusion (BHI) agar plate to ensure the absence of bacterial contamination.

### 2.4 Monitoring the metabolic and motor activity of the parasitic worm *Schistosoma*

#### 2.4.1 Adult Schistosoma preparation

Naval Medical Research Institute (NMRI) mice were infected subcutaneously with approximately 200 *S. mansoni* cercariae. After 7–8 weeks, mice were killed using CO_2_ and dissected. The schistosomes were removed from the hepatic portal system and the mesenteric veins. Worms were washed three times with PBS (pH 7.4), placed in Roswell Park Memorial Institute (RPMI) 1640 culture medium, and kept at 37 °C in an atmosphere of 5% CO_2_ until use. All culture media used were supplemented with 5% inactivated fetal calf serum (iFCS), 100 U/mL penicillin, and 100 μg/mL streptomycin.

Worms were transferred to the stainless steel microcalorimetric vials with 300 μL of the culture medium. The vials were sealed and inserted in the well-plate microcalorimeter according to the manufacturer instructions. The heat production rate calorimetric signal was recorded until it returned to baseline.

#### 2.4.2 Data analysis

Data from measuring channels showing too much electronic noise or poor thermal equilibration were discarded. Growth parameters of planktonic *P. mirabilis* were estimated by fitting a simple exponential model (*Q*_t_ = *Q*_0_ × *e*^(*μ* × *t*)^) to the heat over time curve (obtained by integration of the raw data (i.e., the thermal power over time curve)). The lag phase and the total heat were estimated using the Gompertz model [37]. Growth of hepatocarcinoma microtissues were analyzed by fitting the Gompertz growth model to the heat over time curve. To determine the motor activity of the *Schistsoma* worms, wavelet decomposition was performed according to ref. [17] with the exception that the absolute value of the “noise” (i.e., the random oscillations in the signal resulting form the worm motor activity) is given instead of its amplitude. Statistical comparisons between different groups of data were performed using analysis of variance (ANOVA) after the normality was confirmed using the Shapiro–Wilk normality test. All statistical analyses were performed using the R statistical software and the grofit package [38, 39].

## 3 Results

In all the performed experiments the heat production rate by the living organisms tested was sufficient to produce a detectable signal over time.

**Figure 2:**
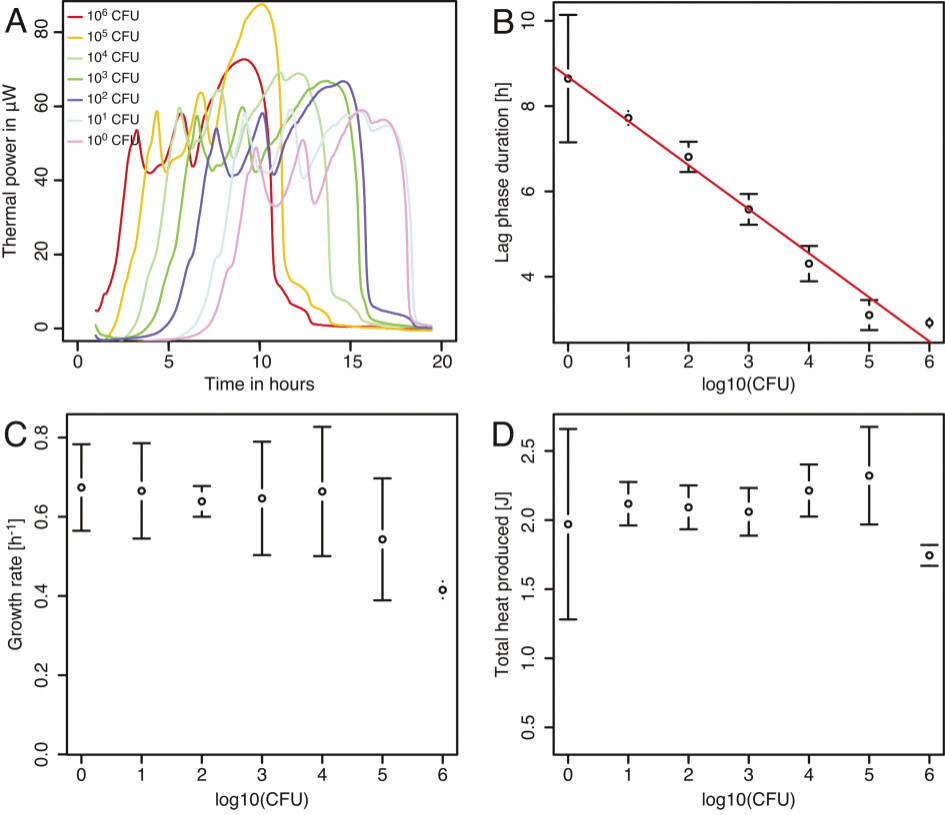
Metabolic thermal power pattern in μW and growth parameter of *P. mirabilis* culture at 37 °C with different inoculum sizes. Cultures were made in 300 μL of LB medium placed in stainless steel calorimetric vials. (A) Representative metabolic thermal power pattern with a cell density ranging from 10^6^ to 10^0^ cfu/mL. (B) Lag phase duration. (C) Growth rate calculated using a simple exponential model. (D) Total heat released over the course of the experiment. Measurements in B, C, D are the mean (dot) and the SD (error bars) of four replicates.

**Figure 3:**
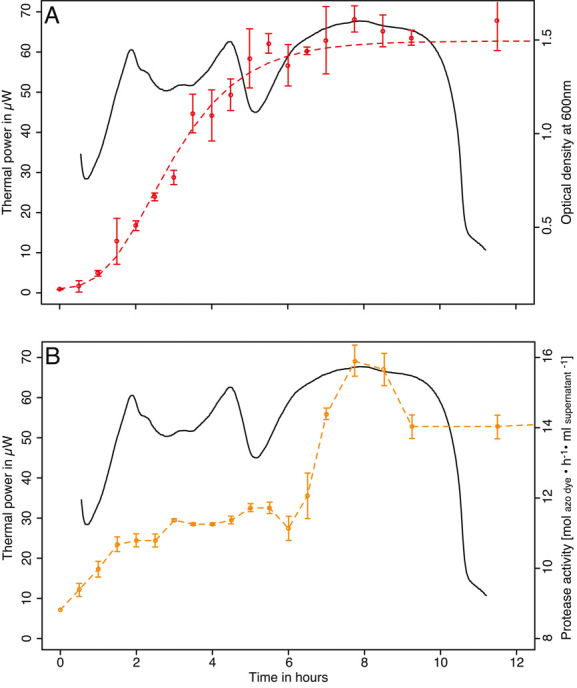
Metabolic thermal power pattern in μW of a 300 μL culture of *P. mirabilis* at 37 °C compared with OD (OD_600_) and protease activity of similar cultures (i.e., same inoculum and same vials) placed in a separate oven at the same temperature. (A) Metabolic thermal power pattern (plain black line) and OD measurements (red dots) fitted with the Gompertz growth model (red line). Average RSD for OD was 8% with a maximum of 28%. (B) Metabolic thermal power pattern (plain black line) and protease activity measured with the azocasein assay (orange dots and line). Average RSD for the azocasein assay was 5% with a maximum of 14%. Metabolic heat production, OD, and protease activity (azocasein assay) were measured in triplicates (dots indicate mean of the three replicates and error bars are the SD). Experiment was repeated twice to confirm the presence of the protease activity peak.

As expected the metabolic heat production during the growth of *P. mirabilis* was easily detected (Fig. 2). Based on the average thermal power of *P. mirabilis* serial dilutions during the 20 min after 1 h of thermal equilibration, the detection limit was estimated to be 3 × 10^4^ cfu per vial. This compares well with other well-known instruments [31]. Slowing down the growth of *P. mirabilis* with chloramphenicol did not significantly change the results and the same detection limit was observed. The raw thermal power signal is composed of three mains peaks. The first two of these peaks are rather sharp as the last one is much wider (Fig. 2A). The maximum thermal power measured was 76 ± 13 μW (*n* = 25). The lag phase duration ranged from 2 to 9 h and was inversely proportional to the inoculum size (Fig. 2B). The growth rate determined was 0.60 ± 0.14 h^–1^ (*n* = 25) (Fig. 2C). The total heat released during the growth experiments was 2.1 ± 0.3 J (*n* = 25) (Fig. 2D). The growth rate measured represent a doubling time of 1.16 h which is in agreement with the OD measurement providing a similar growth curve and a similar growth rate 0.57 h^–1^ (Fig. 3A). When comparing the growth curve obtained using the spectrophotometer we can see that the first two peaks correspond to an increase in cell number. On the contrary the last peak is clearly linked to another metabolic process. Previous observations made with *Escherichia coli* have shown that the first peak can be associated to respiration that is metabolically preferred in facultative anaerobe and that following peaks could be attributed to fermentations of various substrates [19]. Indeed our setup, the amount of oxygen in the medium is ca. 0.08 μmoles and the amount of oxygen contained the headspace (200 μL) of the vial is ca. 1.9 μmoles. Using these values and the Thornton rule [40, 41] we can estimate a total heat of 909 mJ if all oxygen was used. However, after the second peak ca 930 ± 3 mJ have been produced. This shows that even if all oxygen could be used, fermentation would still be needed to provide the remaining heat. Because oxygen consumption rate is much higher than oxygen diffusion rate that is rather slow in aqueous solutions, it is likely that fermentation starts much before oxygen is exhausted from the headspace, thus leading to two peaks of similar sizes. In larger vials oxygen diffusion can be considered as negligible [19]; however, the vials used here have small volume and a reduced oxygen diffusion path. Therefore, oxygen diffusion should be considered [42, 43]. The peak in protease activity determined by our enzyme assay matched the last peak in the metabolic heat production rate curve (Fig. 3B). At this point it should be noted that the protease assay is quite long and that some shift between the protease activity observed using the azocasein assay and the calorimetry data might occur. However, such shift cannot be longer than the assay time (i.e., 1 h). Even considering such shift the two peaks still match well. Furthermore, these observations are consistent with production of large amounts of protease by *P. mirabilis* entering the stationary growth phase as previously reported in the literature [44].

**Figure 4:**
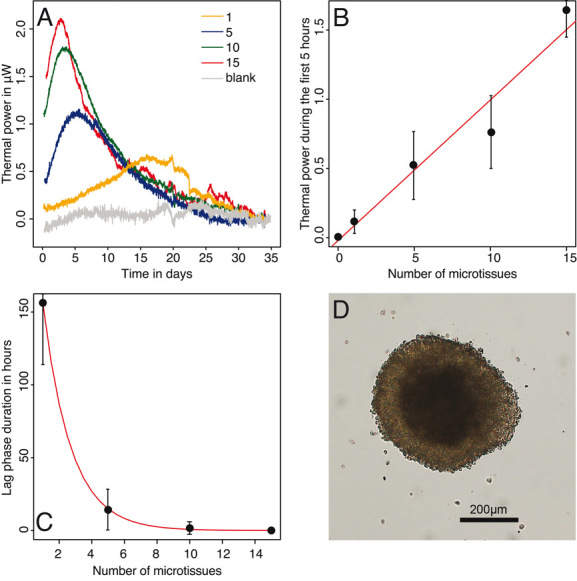
(A) Representative metabolic thermal power pattern in μW of samples containing 1, 5, 10, and 15 hepatocarcinoma microtissues grown in 300 μL of InSphero 3D InSight™ Cell-Culture Media. (B) Average thermal power (proxy for the overall metabolic activity) during the first 5 h of the experiment. Note the linear relationship between the number of microtissues (ca. 1000 cells per microtissue) and the thermal power. (C) Lag phase duration decreasing exponentially as the number of microtissues increases. (D) Picture of a microtissue recovered at the end of the measurements. Measurements in B, C are the mean (dot) and the SD (error bars) of four replicates.

The growth of hepatocarcinoma microtissues released much less heat (Fig. 4A) than bacteria used in this study. The maximum thermal power observed was 2.1 μW. The thermal power measured during the first 5 h of the experiment was directly proportional to the number of microtissues placed in the microcalorimetric vial (Fig. 4B). As a microtissue is made of about 1000 cells (manufacturer data), one can estimate a heat production of 102 pW per cell. In comparison, previous data for rat hepatocytes reported a heat production of 329 pW per cell [30]. Similarly, for bacteria (see above), the lag phase duration that was obtained when fitting the heat over time curve with the Gompertz model also increased as inoculum decreased (Fig. 4C). The measured growth rate was ca. 0.005 h^–1^ which means a doubling time of 138 h. This doubling time seems rather high compared to hepatocarcinoma cell cultures in monolayers where doubling times range from 19 to 66 h [45]; however, it is known that cells proliferate much slower in microtissues [21, 46, 47]. Thus, such doubling time is compatible with microtissue biology. Microscopic observations revealed that at the end of the measurements the microtissues had increased in size from 100 to 150 μm at delivery to 428 ± 71 μm. In addition, they were very cohesive and very few planktonic cells were observed in the medium (Fig. 4D). This indicates that most of the heat comes from the microtissue metabolism. No bacterial contamination was observed, thus indicating that the signal observed can clearly be linked to the metabolic activity of the tumor cells.

**Figure 5:**
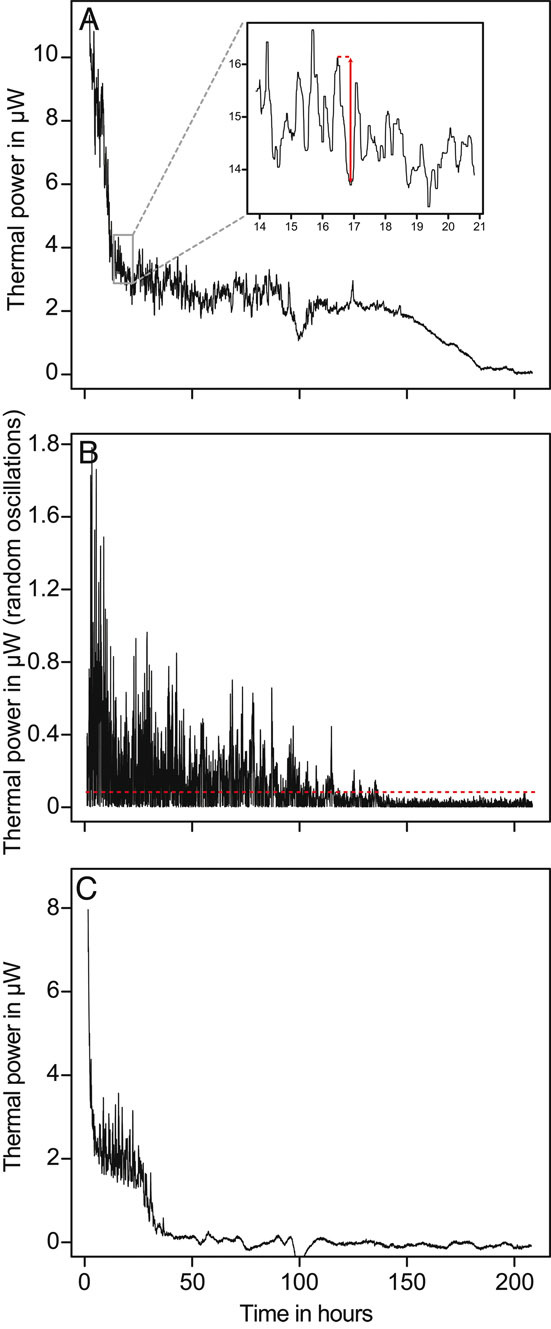
Example of a measurement of *Schistosoma* metabolic and motor activity as recorded by the thermal power in μW. Worms were in stainless steel microcalorimetric vials with 300 μL of RPMI culture medium containing 5% iFCS. (A) Overall signal including metabolic activity and motor activity (additional oscillations in the signal visible in the insert) of one vial containing five worms. (B) Random oscillations used as a proxy for the motor activity. (C) Overall signal including metabolic activity and motor activity of one vial containing one female *Schistosoma*.

Finally, the *Schistoma* showed decreasing metabolic activity over time. The overall activity decreased rapidly over the first 10–24 h. Then, the overall metabolic activity stabilized for a time period lasting between a few hours and a few days (up to 180 h) and then returned to baseline indicating worm death (Fig. 5A). This overall pattern is in close agreement with the pattern previously observed [17]. On the other hand the worms' motor activity was clearly visible and followed a decreasing trend over time as well. Random oscillations (i.e., the additional “noise” in the signal resulting form the worm motor activity) showed a maximum of 1.8 μW at the beginning (Fig. 5B) and decreased below detection limit slightly before the metabolic activity returned to baseline consistent with senescence and death. Considering an electronic noise of ca. 50 nW the maximal S/N is ca. 35:1. The motor activity signal was very strong even when a single worm was considered (Fig. 5C).

## 4 Discussion

In all the applications tested the isothermal microcalorimeter with its well-plate format sample holder provided reproducible data. For bacteria (i.e., *P. mirabilis*) the growth rate and the lag phase duration can be determined using a microcalorimeter and data are consistent with conventional OD readings and CFU counts. Potentially, the determined growth rate can be used to assess the effect of a substance on microbial growth, and the lag phase duration can be used to estimate the bacterial load in a sample or a potential bactericidal effect more quickly and as reliably than time consuming conventional methods used for screening [6, 37]. This instrument could also be used for opaque samples (blood, stool, or milk) where optical methods (i.e., OD measurement) could not be applied.

For microtissues and parasitic worms, our data are in agreement with previously collected data using other microcalorimeters and with data available in the literature [17, 48, 49]. For both microtissues and parasitic worms microscopy remains the gold standard; however, our results demonstrates that a well-plate format microcalorimeter is sensitive enough to offer a similar throughput at a much lower workload. In addition, combining microcalorimetric data with other sets of data allows pinpointing of interesting time points in the heat production profile. For example, with *P. mirabilis* the last peak reflects the protease activity and could be used to assess production of such enzymes in other organisms as well. This peak is quite marked since the enthalpy of hydrolysis of peptide bonds is high [50, 51], although this also depends on the solution pH and buffer used. As a result the technique is sensitive enough and it opens promising research avenues in the screening of protease inhibitors, as those enzymes have often been implicated as a virulence factor of bacteria [52]. Furthermore, considering the importance of protease inhibitors in several other infections including human immunodeficiency virus (HIV), hepatitis, malaria, Giardiasis, and Chagas diseases as well as cancer [53–56] one might hypothesize that a rapid calorimetric screening of protease inhibitors could easily be implemented, thus allowing higher throughput and faster screening in drug development.

Similarly when considering cancer, it has been clearly demonstrated over the last years that 3-D microtissue models have a higher predictability for in vivo response than conventional monolayer culture [57]. Our results show that overall viability and growth of cancer microtissues can be easily monitored over time without the need of disrupting them to perform a viability assay. Considering the high intratumor genetic heterogeneity [58], phenotypic screening of the susceptibility of tumors to antitumor agents potentially provides a cheap alternative for selecting the best possible chemotherapy and aids the development of personalized medicine based on tumor biopsies. Previous work on urogenital tumor biopsies have indeed demonstrated that microcalorimetry can be used as an accurate diagnostic tool since cancerous tissues produce more heat than healthy tissues. In addition it was shown that microcalorimetric measurements performed on such biopsies could be related to the histological grading of the same biopsies [22].

Finally with respect to applications in parasitology, our results indicate that the low mass and the high thermal conductivity of the microcalorimetric vials contribute to an increased sensitivity compared to other instruments. Thus, it becomes possible to get accurate data with single worms decreasing the need to sacrifice hosts. Furthermore, the workload is rather low since there is no need for continual microscopic evaluation (mostly based on the motor activity) to assess the viability of worm, as well as their senescence and death.

Although, the well plate format is extremely common for biological assays, they often require labeled substrate or labeled organisms. On the other hand isothermal microcalorimetry is label-free and has become more commonly used among biologists and physicians over the last few years [1, 19]. Here, we have demonstrated that a well plate instrument combining the throughput of well-plate assays and the sensitivity of isothermal microcalorimetry can be easily used in the fields of microbiology, oncology, and parasitology. The label free and passive nature of isothermal microcalorimetry allows making measurements on complex 3D structures (such has microtissues) that would otherwise require destruction to perform a conventional assay (DNA or protein quantification for example). Therefore, we believe that there is not only room, but also a bright future for this technique in applied biology and medicine. Future users will need to adapt current assays or develop new ones and optimize those in their own fields of research. Particular attention should be paid to the interpretation of the calorimetric signal and its “translation” into a biologically meaningful equivalent. For example the choice of the growth model applied to the bacterial growth needs to be appropriate [37] and one must take care that the fitted part of the calorimetric curve actually corresponds to the growth. Similarly, the wavelet decomposition used to extract the worms' motor activity needs to be optimized depending on the type of worms considered and the experimental conditions. Only after careful model choice and optimization can the method be applied routinely and data analyzed automatically by batches. Such potential for automation of the data treatment per batches (i.e., in this case a well-plate) also contribute to the attractiveness of the technique.

## Abbreviations

ANOVA: analysis Of variance
ATCC: American Type Culture Collection
BHI: brain heart infusion
CFU: colony forming unit
DSMZ: Deutsche Sammlung von Mikroorganismen und Zellkulturen
iFCS: inactivated fetal calf serum
LB: Luria Broth
NMRI: Naval Medical Research Institute
PBS: phosphate buffered saline
RPMI: Roswell Park Memorial Institute
RSD: relative standard deviation

## References

[1] Wadsö I (2002). Isothermal microcalorimetry in applied biology. Thermochim. Acta.

[2] Bonkat G, Braissant O, Widmer AF, Frei R (2012). Rapid detection of urinary tract pathogens using microcalorimetry: Principle, technique and first results. BJU Int.

[3] Borens O, Yusuf E, Steinrucken J, Trampuz A (2013). Accurate and early diagnosis of orthopedic device-related infection by microbial heat production and sonication. J. Orthop. Res.

[4] Trampuz A, Steinhuber A, Wittwer M, Leib SL (2007). Rapid diagnosis of experimental meningitis by bacterial heat production in cerebrospinal fluid. BMC Infect. Dis.

[5] Baldoni D, Hermann H, Frei R, Trampuz A, Steinhuber A (2009). Performance of microcalorimetry for early detection of methicillin resistance in clinical isolates of Staphylococcus aureus. J. Clin. Microbiol.

[6] Braissant O, Muller G, Egli A, Widmer A (2014). Seven hours to adequate antimicrobial therapy in urosepsis using isothermal microcalorimetry. J. Clin. Microbiol.

[7] Li X, Liu Y, Zhao R, Wu J (2000). Microcalorimetric study of Escherichia coli growth Inhibited by the selenomorpholine complexes. Biol. Trace Elem. Res.

[8] Tan A-M, Lu J-H (1999). Microcalorimetric study of antiviral effect of drug. J. Biochem. Biophys. Methods.

[9] Xi L, Yi L, Jun W, Huigang L, Songsheng Q (2002). Microcalorimetric study of Staphylococcus aureus growth affected by selenium compounds. Thermochim. Acta.

[10] Yang LN, Xu F, Sun XN, Zhao ZB, Song GC (2008). Microcalorimetric studies on the action of different cephalosporins. J. Therm. Anal. Calorim.

[11] Galindo FG, Rocculi P, Wadso L, Sjohlm I (2005). The potential of isothermal calorimetry in monitoring and predicting quality changes during processing and storage of minimally processed fruits and vegetables. Trends Food Sci. Technol.

[12] Kabanova N, Stulova I, Vilu R (2012). Microcalorimetric study of the growth of bacterial colonies of Lactococcus lactis IL1403 in agar gels. Food Microbiol.

[13] Kabanova N, Stulova I, Vilu R (2013). Microcalorimetric study of growth of Lactococcus lactis IL1403 at low glucose concentration in liquids and solid agar gels. Thermochim. Acta.

[14] Wadso L, Galindo FG (2009). Isothermal calorimetry for biological applications in food science and technology. Food Control.

[15] Doostmohammadi A, Monshi A, Fathi MH, Karbasi S (2011). Direct cytotoxicity evaluation of 63S bioactive glass and bone-derived hydroxyapatite particles using yeast model and human chondrocyte cells by microcalorimetry. J. Mater. Sci. Mater. Med.

[16] Keiser J, Manneck T, Kirchhofer C, Braissant O (2013). Isothermal microcalorimetry to study the activity of triclabendazole and its metabolites on juvenile and adult Fasciola hepatica. Exp. Parasitol.

[17] Manneck T, Braissant O, Ellis W, Keiser J (2011). Schistosoma mansoni: antischistosomal activity of the four optical isomers and the two racemates of mefloquine on schistosomula and adult worms in vitro and in vivo. Exp. Parasitol.

[18] Tritten L, Braissant O, Keiser J (2012). Comparison of novel and existing tools for studying drug sensitivity against the hookworm Ancylostoma ceylanicum in vitro. Parasitology.

[19] Braissant O, Wirz D, Gopfert B, Daniels AU (2010). Use of isothermal microcalorimetry to monitor microbial activities. FEMS Microbiol Lett.

[20] Charlebois SJ, Daniels AU, Lewis G (2003). Isothermal microcalorimetry: An analytical technique for assessing the dynamic chemical stability of UHMWPE. Biomaterials.

[21] West AR, Zaman N, Cole DJ, Walker MJ (2013). Development and characterization of a 3D multicell microtissue culture model of airway smooth muscle. Am. J. Physiol.-Lung C.

[22] Kallerhoff M, Karnebogen M, Singer D, Dettenbach A (1996). Microcalorimetric measurements carried out on isolated tumorous and nontumorous tissue samples from organs in the urogenital tract in comparison to histological and impulse cytophotometric investigations. Urol. Res.

[23] Bluthnerhassler C, Karnebogen M, Schendel W, Singer D (1995). Influence of malignancy and cyctostatic treatment on microcalorimetric behavior of urological tissue samples and cell-cultures. Thermochim. Acta.

[24] Karnebogen M, Singer D, Kallerhoff M, Ringert RH (1993). Microcalorimetric investigations on isolated tumorous and nontumorous tissue samples. Thermochim. Acta.

[25] Kemp, R. B., *Handbook of Thermal Analysis and Calorimetry: From Macromolecules to Man*

[26] Montagnes D, Roberts E, Lukes J, Lowe C (2012). The rise of model protozoa. Trends Microbiol.

[27] Wadso I, Goldberg RN (2001). Standards in isothermal microcalorimetry (IUPAC technical report). Pure Appl. Chem.

[28] Wadso L (2010). Operational issues in isothermal calorimetry. Cement Concr. Res.

[29] James, M. A., *Thermal and Energetic Studies of Cellular Biological Systems*

[30] Kemp RB, Guan YH (2000). The application of heat flux measurements to improve the growth of mammalian cells in culture. Thermochim. Acta.

[31] Braissant O, Wirz D, Gopfert B, Daniels AU (2010). Biomedical use of isothermal microcalorimeters. Sensors (Basel).

[32] Lerchner J, Maskow T, Wolf G (2008). Chip calorimetry and its use for biochemical and cell biological investigations. Chem. Eng. Process.

[33] Maskow T, Lerchner J, Peitzsch M, Harms H, Wolf G (2006). Chip calorimetry for the monitoring of whole cell biotransformation. J. Biotechnol.

[34] Maskow T, Schubert T, Wolf A, Buchholz F (2011). Potentials and limitations of miniaturized calorimeters for bioprocess monitoring. Appl. Microbiol. Biot.

[35] Buchholz F, Harms H, Maskow T (2010). Biofilm research using calorimetry – a marriage made in heaven?. Biotechnol. J.

[36] Mariana F, Buchholz F, Lerchner J, Neu TR (2013). Chip-calorimetric monitoring of biofilm eradication with antibiotics provides mechanistic information. Int. J. Med. Microbiol.

[37] Braissant O, Bonkat G, Wirz D, Bachmann A (2013). Microbial growth and isothermal microcalorimetry: Growth models and their application to microcalorimetric data. Thermochim. Acta.

[38] Kahm M, Hasenbrink G, Lichtenberg-Frate H, Ludwig J, Kschischo M (2010). grofit: Fitting biological growth curves with R. J. Stat. Softw.

[39] R Core Team, *R: A Language and Environment for Statistical Computing*

[40] Cordier JL, Butsch BM, Birou B, Vonstockar U (1987). The relationship between elemental composition and heat of combustion of microbial biomass. Appl. Microbiol. Biot.

[41] Hansen LD, Criddle RS, Battley EH (2009). Biological calorimetry and the thermodynamics of the origination and evolution of life. Pure Appl. Chem.

[42] Maskow T, Morais FM, Rosa LFM, Qian YG, Harnisch F (2014). Insufficient oxygen diffusion leads to distortions of microbial growth parameters assessed by isothermal microcalorimetry. RSC Adv.

[43] Zaharia DC, Muntean AA, Popa MG, Steriade AT (2013). Comparative analysis of Staphylococcus aureus and Escherichia coli microcalorimetric growth. BMC Microbiol.

[44] Bonato MCM, Costa SOP, Bianco M (1982). Regulation of extracellular protease secretion in proteus-mirabilis. Rev. Bras. Genet.

[45] Domenis R, Bisetto E, Rossi D, Comelli M, Mavelli I (2012). Glucose-modulated mitochondria adaptation in tumor cells: A focus on ATP synthase and inhibitor factor 1. Int. J. Mol. Sci.

[46] Ceresa CC, Knox AJ, Johnson SR (2009). Use of a three-dimensional cell culture model to study airway smooth muscle-mast cell interactions in airway remodeling. Am. J. Physiol.-Lung C.

[47] Song J, Rolfe BE, Hayward IP, Campbell GR, Campbell JH (2000). Effects of collagen gel configuration on behavior of vascular smooth muscle cells in vitro: Association with vascular morphogenesis. In Vitro Cell Dev.-Anim.

[48] Kelm JM, Sanchez-Bustamante CD, Ehler E, Hoerstrup SP (2005). VEGF profiling and angiogenesis in human microtissues. J. Biotechnol.

[49] Sanchez-Bustamante CD, Kelm JM, Mitta B, Fussenegger M (2006). Heterologous protein production capacity of mammalian cells cultivated as monolayers and microtissues. Biotechnol. Bioeng.

[50] Rawitscher M, Wadsö I, Sturtevant JM (1961). Heats of hydrolysis of peptide bonds1. J. Am. Chem. Soc.

[51] Sturtevant JM (1953). Heats of hydrolysis of amide and peptide bonds1. J. Am. Chem. Soc.

[52] Ingmer H, Brondsted L (2009). Proteases in bacterial pathogenesis. Res. Microbiol.

[53] Andrews KT, Fairlie DP, Madala PK, Ray J (2006). Potencies of human immunodeficiency virus protease inhibitors in vitro against Plasmodium falciparum and in vivo against murine malaria. Antimicrob. Agents Chemother.

[54] Doyle PS, Zhou YM, Engel JC, McKerrow JH (2007). A cysteine protease inhibitor cures Chagas' disease in an immunodeficient-mouse model of infection. Antimicrob. Agents Chemother.

[55] Dunn LA, Andrews KT, McCarthy JS, Wright JM (2007). The activity of protease inhibitors against Giardia duodenalis and metronidazole-resistant Trichomonas vaginalis. Int. J. Antimicrob Agents.

[56] Pyrko P, Kardosh A, Wang W, Xiong W (2007). HIV-1 protease inhibitors nelfinavir and atazanavir induce malignant glioma death by triggering endoplasmic reticulum stress. Cancer Res.

[57] Håkanson M, Cukierman E, Charnley M (2014). Miniaturized pre-clinical cancer models as research and diagnostic tools. Adv. Drug Deliv. Rev.

[58] Gerlinger M, Rowan AJ, Horswell S, Larkin J (2012). Intratumor heterogeneity and branched evolution revealed by multiregion sequencing. New Engl. J. Med.

